# Understanding Protein Protocadherin-19 (PCDH19) Syndrome: A Literature Review of the Pathophysiology

**DOI:** 10.7759/cureus.25808

**Published:** 2022-06-10

**Authors:** Juan A Moncayo, Ivan N Ayala, Jennifer M Argudo, Alex S Aguirre, Jashank Parwani, Ana Pachano, Diego Ojeda, Steven Cordova, Maria Gracia Mora, Christiany M Tapia, Juan Fernando Ortiz

**Affiliations:** 1 Neurology, Pontificia Universidad Católica del Ecuador, Quito, ECU; 2 Endocrinology, Mayo Clinic, Rochester, USA; 3 Child Neurology, Universidad de Cuenca, Cuenca, ECU; 4 Medicine, Universidad San Francisco de Quito, Quito, ECU; 5 Neurology, Lokmanya Tilak Municipal Medical College, Mumbai, IND; 6 Medicine, Universidad de las Americas, Quito, ECU; 7 Neurology, Universidad San Francisco de Quito, Quito, ECU; 8 Neurology, Caduceus Healthcare, Miami, USA; 9 Surgery, Universidad de Cuenca, Cuenca, ECU

**Keywords:** gaba r dysfunciton, cellular interference, blood brain barrier, refractory epilepsy, clusters, epilepsy, pcdh19

## Abstract

PCDH19 syndrome is a monogenic epilepsy related to the protein protocadherin-19 (PCDH19) gene, which encodes for a protein important for brain development. The protein also seems to regulate gamma-aminobutyric acid type A receptors (GABA(A)(R)). The disease presents with refractory epilepsy that is characterized by seizures occurring in clusters. Till now, the pathophysiology of the disease is mainly unknown, so we conducted a literature review to elucidate the pathophysiology of PCDH19-related epilepsy. We used two databases to investigate this literature review (Google Scholar and PubMed). We selected full-text papers that are published in the English language and published after the year 2000. We selected initially 64 papers and ended up with 29 to conduct this literature review. We found four main theories for the pathophysiology of PCDH19-related epilepsy: GABA(A)(R) dysregulation, blood-brain barrier (BBB) dysfunction, cellular interference, and the AKR1C1-3 gene product deficiency.

GABA(A)(R) dysfunction and expression cause decreased effective inhibitory currents predisposing patients to epilepsy. BBB dysfunction allows the passage of methyl-D-aspartate (NMDA)-type glutamate receptor antibodies (abs-NR) through the BBB susceptible membrane. The cellular interference hypothesis establishes that the mutant and non-mutant cells interfere with each other’s communication within the same tissue. Women are more susceptible to being affected by this hypothesis as men only have one copy of the x gene and interference is mediated by this gene, meaning that it cannot occur in them. Finally, downregulation and deficiency of the AKR1C3/AKR1C2 products lead to decreasing levels of allopregnanolone, which diminish the regulation of GABA(A)(R).

## Introduction and background

PCDH19 syndrome is a monogenic epilepsy related to PCDH19, which encodes for a protein important for brain development. This protein also seems to regulate the GABA-A receptor.

The PCDH19 gene is located on chromosome Xq22.1; it consists of six exons and encodes for a calcium-dependent adhesion molecule called protein protocadherin-19 (PCDH19). The protein is expressed mainly in the central nervous system, the limbic system, and the cortex [1]. This protein is involved in signal transduction at the synapses. The protein has an extracellular domain, six conserved cadherin repeats that mediate fusion, a transmembrane region, and an intracellular C-terminal domain [1]. Pathogenic variants of the gene affect mainly the exon [1].

The main clinical feature of PCHD19-related epilepsy is refractory epilepsy. Seizures in these patients tend to be triggered by photosensitivity. The seizures usually occur in clusters. However, there is no definite occurrence of cluster seizures in these patients [2]. PCDH19-associated seizures tend to be tonic or tonic-clonic types, which are brief and frequently cluster over hours to days before stopping, often refractory to treatment. The seizures in PCDH19 can be tonic-clonic, atonic, myoclonic, absent, or occurring in a cluster [3]. The onset of seizures is between the ages of 10 and 14 months and may decrease or completely remit in adolescence. Developmental delay is seen in 85% of the patients. The condition is X-linked, so it affects mainly female patients [4].

Other symptoms include delayed development, behavior dysregulation, and intellectual disability. The most commonly associated behavioral abnormalities are autistic spectrum disorder, general anxiety disorder, and psychosis [5]. Currently, the pathophysiology of the disease is mainly unknown. This literature review will give a better understanding of this disorder and the pathophysiological basis of PCDH19-related epilepsy.

## Review

Methods

Studies were selected by applying the following exclusion/inclusion criteria: (1) full-text papers, (2) papers published in the English language, and (3) papers published after 2000. The exclusion criteria are as follows: (1) only abstracts, (2) papers not published in the English language, and (3) papers published before the year 2000. Additional studies were included via reference lists of identified papers and related articles encountered in Google Scholar and PubMed.

Search Terms

("PCDH19"[Title/Abstract] AND "Molecular"[Title/Abstract]) OR ("PCDH19"[Title/Abstract] AND "Receptor"[Title/Abstract]) OR ("AKR1C1-3"[Title/Abstract] AND "PCDH19"[Title/Abstract]) OR ("blood brain barrier"[Title/Abstract] AND "PCDH19"[Title/Abstract]) OR ("gaba receptor"[Title/Abstract] AND "PCDH19"[Title/Abstract]) OR ("PCDH19"[Title/Abstract] AND "INTERFERENCE"[Title/Abstract])

Results

After using the search terms, 64 papers were drawn; by following the inclusion and exclusion criteria, we ended up with 26 papers to include in the analysis of the pathophysiological review of PCDH19.

Discussion

The PCDH19 gene is located on chromosome Xq22.1, made of six exons encoding for a calcium-dependent adhesion molecule called protein protocadherin-19 (PCDH19). So far, more than 175 pathogenic variants have been described, with no clear phenotype/genotype correlation. Thus, any of the pathogenic variants are believed to cause a loss of function of PCDH19 [6]. This condition follows a unique X-linked fashion, in which females exhibit symptoms while carrier males do not [7]. Recently, there has been an increase in the number of diagnosed cases as the recognition of the gene and the spectrum of phenotypic manifestations have grown that involves early-onset epilepsy, intellectual disability (ID), and sporadic focal epilepsy [8,9].

The PCDH19 protein is involved in critical functions such as the neuronal proliferation of progenitor cells, cell adhesion, synaptic transmission, and neuronal migration. In our review, we found that the dysfunction of PCDH19-related epilepsy might be due to four causes: The GABA receptor (GABA(A)R) hypothesis, blood-brain barrier (BBB) dysfunction hypothesis, AKR1C1-3 gene hypothesis, and the cellular interference hypothesis. Each hypothesis is reviewed below.

GABA Receptor Hypothesis

The PCDH19 protein interacts with gamma-aminobutyric acid type A receptors (GABA(A)R) [6,10]. During brain development, GABAergic signaling induces differentiation of neuronal progenitors, neuronal migration, and maturation. This GABA(A) role function occurs before the GABAergic "switch" to gaining its inhibitory quality, and its dysfunction may be a key factor in the broad cognitive and neuropsychiatric symptoms in PCDH19 syndrome [11].

A decrease in the GABA(A)R function, and possibly also expression, might be the cause of epilepsy. PCDH19 mutation in rats has been shown to affect the GABA(A)-mediated transmission, and PCDH19 binding might directly affect the GABA(A)R kinetics [6]. On the other hand, Bassani et al. observed reduced expression of GABA(A)R on the surface of rat hippocampal neurons expressing downregulation of PCDH19 [11]. Mazzoleni et al. proposed that GABA(A)R signaling plays a key role in the development of PCDH19-related epilepsy, adding the syndrome to the demonstrated list of disorders with a malfunction of this receptor including Dravet syndrome, fragile X syndrome, Rett syndrome, and Prader-Willi syndrome, showing that a dysfunction in these receptors leads to pathological outcomes [1,6].

BBB Dysfunction Hypothesis

PCDH19 seems to be highly concentrated in the brain. Daneman et al. tested the expression of PCDH19 in rats and found that this gene has a higher expression of PCHH19 in the microvascular endothelial cells of the brain than in the liver or lungs. So, if PCDH19 is highly expressed in brain microvascular endothelial cells, an impairment of the protein may cause BBB vulnerability [12-14].

The expression of PCDH19 may be altered in the BBB when inflammation occurs. The BBB alteration during inflammation is predisposed to the recurrence of seizures of PCDH19-related epilepsy [13]. According to the author, a study by Higurashi et al. showed that corticosteroids effectively controlled the cluster seizures and another acute symptom in these patients, possibly due to restoration of the BBB [12].

Higurashi et al. also found high levels of N-methyl-D-aspartate (NMDA)-type glutamate receptor (abs-NR) in the serum or cerebrospinal fluid (CSF); Abs-NR could pass the BBB because it is susceptible in patients with PCDH19-related epilepsy. Besides restoring the BBB, corticosteroids might decrease the levels of antibodies in these patients [12].

AKR1C1-3 Gene Hypothesis

The aldo-keto reductase 1C (AKR1C1) family has four types of genes. In this family, AKR1C1-3 is the only one found in the brain. While AKR1C2 is not in, the brain interacts with AKR1C1-3. These genes (AKR1C2 and AKR1C3) seem to be important in the pathophysiology of PCDH19-related epilepsy. The product of AKR1C3/AKR1C2 converts 5-alpha-dihydro-progesterone to allopregnanolone, the most potent positive modulator of GABA(A)R that increases the tonic and phasic inhibitory currents of the receptor [15-17].

AKR1C1-3 seems to be key in the pathophysiology of PCDH19-related epilepsy. Tan et al. found that females have lower levels of AKR1C1-3 and downregulation of AKR1C2. The deficiency of AKR1C1-3 and the downregulation of AKR1C2 decrease the levels of allopregnanolone, so the GABA(A)R in these patients is less regulated [14].

The discovery of the alteration in steroidogenesis could help guide the treatment of this unique disease, probably also explaining the poor response to anticonvulsants and the purpose of corticoids validated by Higurashi et al. The link between epilepsy and fluctuations in sex steroid hormones has been made in the past as described by Reddy et al. in catamenial epilepsy, where a high ratio between estradiol:progesterone in the perimenstrual period increases seizure activity [15]; this association is also seen in female patients with PCHD19 [16,17]. Allopregnanolone is known to have anticonvulsive properties [18], which is why the use of ganaxolone, a synthetic analog, has gained clinical relevance showing promising results not only in mouse knockout models for fragile X syndrome [17] but also in adults and children [19-21].

Cellular Interference Hypothesis

Abnormal communication between "mutated" cells and "normal" cells would result in a disarranged functioning [22]. The term "cellular interference" was taken from the "metabolic interference" model developed by Johnson in 1980 and newly used in this context by Wieland et al. in 2004 [22].

Johnson proposed that mutated females, as well as mosaic males, would be affected. Hemizygous males are clinically asymptomatic. This implies that the losing function of protocadherin 19 does not correspondingly involve pathogenicity; it seems to be that other proteins can compensate for the loss in humans. In contrast, heterozygous women present with epileptic encephalopathy [22]. The PCDH19 gene lies in a region that can succumb to X inactivation leading to tissue mosaicism; the latter involves cells that either present with inactivated mutated PCDH19 allele that expresses normal protein or negative PCDH19 that has inactivated its normal allele. Due to this mutation, there is cellular interference expressed by loss of function at the cell level and gain of function at the tissue level [22,23]. Nonetheless, for Johnson's theory to be correct, it is necessary to demonstrate that homozygous females are unaffected like hemizygous males.

Cellular interference is not unique to PCDH19-related epilepsy. For example, craniofrontonasal syndrome (CFNS) is caused by a mutation in the gene EFNB1, which encodes for Ephrin B1 [23], a protein whose signaling plays a role in pattern formation and cell migration during the developmental period [24,25].

Additional Theories Based on Animal Models

Emond et al. explored the functioning of PCDH19 using zebrafish [25]. The ortholog of PCDH19 in zebrafish has shown its crucial importance in brain morphogenesis, when subjected to partial depletion of this protein with morpholino oligonucleotides; the cell migration in the anterior neural plate was stagnated, where PCDH19 is expressed [26]. Another interesting finding made by Biswas et al. demonstrates the interaction in vivo and in vitro of N-cadherin and PCDH19, supporting the hypothesis that PCDH19 is a member of the cadherins; it also regulates cell adhesion, synapse formation, and neuronal migration [26]. Further development of animal models is needed as an important step forward in elucidating these observations [27].

Even though the mammalian genome contains a broad range of PCDH genes, a few have been linked to human pathology. So is the case of PCDH15, which causes Usher syndrome and PCDH19. It has been proposed by several authors that defects in these genes are also related to neurodevelopmental disorders such as autism, schizophrenia, and mental retardation [28,29].

## Conclusions

The GABA receptor hypothesis is based on the interaction between PCDH19 mutations and the effect on the GABA-A-R function. The GABA-A-R may play a role in early neural development, before acquiring its inhibitory properties. Dysfunction of this receptor would embody the clinical symptoms of PCDH19 syndrome. BBB alteration might increase the susceptibility of these patients and allow the passage of Abs-NR, which could worsen the symptoms. Corticosteroids may ameliorate the symptoms by decreasing their migration and restoring BBB permeability.

The cellular interference theory establishes that within the same tissue, there is a coexistence of two types of cells: normal cells and mutated cells that interact and interfere with each other, causing an imbalance. Downregulation and deficiency of the AKR1C3/AKR1C2 products lead to decreasing levels of allopregnanolone, which diminish GABA-A-R regulation. On the other hand, high doses of allopregnanolone have shown anticonvulsive properties. It is possible that a combination of all or some of these hypotheses plays a role in the pathophysiology of PCHD19 syndrome, with some contributions to various degrees.
